# Expression and clinical significance of S100A8/9 in adults with secondary phagocytic lymphohistiocytosis

**DOI:** 10.3389/fmolb.2025.1607352

**Published:** 2025-07-15

**Authors:** Ziwei Fang, Xin Gao, Limin Duan, Jujuan Wang, Tian Tian, Ji Xu, Yongqian Shu, Guangli Yin, Hongxia Qiu

**Affiliations:** ^1^ Department of Hematology, The First Affiliated Hospital of Nanjing Medical University, Jiangsu Province Hospital, Nanjing, China; ^2^ Department of Oncology, The First Affiliated Hospital of Nanjing Medical University, Jiangsu Province Hospital, Nanjing, China

**Keywords:** phagocytic lymphohistiocytosis, S100A8/9, RCS, diagnosis, prognosis

## Abstract

**Introduction:**

The study aimed to investigate the diagnostic and prognostic value of serum S100A8/9 levels with sHLHa, a high-mortality multiorgan inflammatory syndrome with no reliable clinical biomarkers, where calreticulin’s role is unclear.

**Methods:**

This was a study of 67 newly diagnosed sHLHa patients. 48 patients met criteria and were analyzed. ELISA detected S100A8/9 levels in patients and controls. The optimal classification threshold for S100A8/9 was determined to be 2.44 µg/mL by restricted cubic spline (RCS) curve analysis. Patients were categorized. Correlations, diagnostic efficacy, survival differences, and prognosis impacts were analyzed.

**Results:**

Serum S100A8/9 levels in sHLHa patients were greater than in healthy controls. Various analyses showed its diagnostic and prognostic value. ANC<1.0 × 10^9^/L and high S100A8/9 expression group were independent risk factors for poor prognosis in patients with sHLHa. It’s correlated with liver function indicators and HScore.

**Discussion:**

This study evaluates S100A8/9 in sHLHa diagnosis and prognosis. S100A8/9 levels are useful for differentiating patients, providing etiologic and survival info. They show a nonlinear positive correlation with survival and a threshold effect. Serum S100A8/9 levels offer potential biomarkers, and further studies are needed.

## Introduction

Secondary hemophagocytic lymphohistiocytosis in adults (sHLHa) is a multiorgan inflammatory response syndrome triggered by an abnormal inflammatory response that further results in the aggregation of activated T lymphocytes and macrophages in multiple organs and the outbreak of a cytokine stor (Valade et al., 2021; Nguyen et al., 2024). The disease is usually triggered by a variety of causative factors, such as infections, malignancies, and rheumatologic-immune disorders (Griffin et al., 2020). Patients with untreated sHLHa have a high mortality rate (20%–53%, up to 70% for specific types of patients) (La Rosée et al., 2019; Rouphael et al., 2007). The main clinical features of sHLHa include persistent fever, hepatosplenomegaly, hematopenia in more than two lineages of cells, methemoglobinemia, and phagocytic phenomena. Commonly used clinical diagnostic indicators (e.g., ferritin, lactate dehydrogenase, and the splenic index) are insufficient to achieve early diagnosis and prognostic assessment of the disease (Henter, 2025). In addition, there is no single biomarker that reliably predicts disease progression and patient prognosis (Wu et al., 2024). This current situation highlights the importance and urgency of finding novel biomarkers to improve the diagnosis and prognostic assessment of sHLHa.

In recent years, S100A8 and S100A9 have been shown to be constitutively expressed in myeloid cells as endogenous alert proteins and stored as heterodimeric complexes (S100A8/9) in granules (Leukert et al., 2006). S100A8/9 is highly correlated with monocyte macrophage activation in hyperinflammatory responsive diseases, autoimmune disorders, and a variety of tumors (Manfredi et al., 2023; Muñoz-Grajales et al., 2023; Perego et al., 2020; Wang Q. et al., 2023; Yao et al., 2022) and is able to promote the production of inflammatory factor (Wen et al., 2018). In the pathology of sHLHa, the uncontrolled inflammatory cascade, the ensuing cytokine storm and immunosuppression are the main drivers of disease progression and poor prognosis (Nguyen et al., 2024). However, it is unclear whether elevated S100A8/9 levels are associated with poor survival in sHLHa patients, especially when independent from traditional risk factors (e.g., age and underlying disease).

Based on the above research background and gaps in the existing knowledge, the aim of this study was to systematically evaluate the clinical value of serum S100A8/9 levels in the diagnosis and prognostic assessment of patients with sHLHa.

## Patients and methods

### Patients

In this study, 67 patients with a primary diagnosis of sHLHa diagnosed from January 2022 to December 2024 in the Department of Hematology of the First Affiliated Hospital of Nanjing Medical University were retrospectively analyzed. On the basis of the inclusion and exclusion criteria, 48 patients with sHLHa were ultimately included. The diagnostic criteria for sHLHa refer to the HLH-2004 (Henter et al., 2007) and HScore scoring criteria (Fardet et al., 2014). The inclusion criteria included (Valade et al., 2021) age ≥18 years and (Nguyen et al., 2024) fulfillment of 5 or more of the 8 HLH-2004 diagnostic criteria. The exclusion criteria included (Valade et al., 2021) age <18 years, (Nguyen et al., 2024), history of cirrhosis or other severe liver disease, and (Griffin et al., 2020) pregnancy status. The study was approved by the Ethics Committee of the First Affiliated Hospital of Nanjing Medical University (Clinical Trial Registration No. ChiCTR2000032421) and was conducted in strict accordance with the requirements of the Declaration of Helsinki. All patients signed an informed consent form to allow medical record review.

### Clinical data

Retrospective analysis of the patients’ admission data revealed the following clinical characteristics and laboratory findings: (Valade et al., 2021) age and sex; (Nguyen et al., 2024); clinical manifestations: fever and hepatosplenomegaly; and (Griffin et al., 2020) laboratory tests: complete blood count, fibrinogen (FIB), triglyceride (TG), alanine aminotransferase (ALT), aspartate aminotransferase (AST), lactate dehydrogenase (LDH), alpha-hydroxybutyrate dehydrogenase (α-HBDB), adenosine deaminase (ADA), serum ferritin, and serum soluble interleukin-2 receptor (sIL-2R, sCD25) levels. Due to the limited equipment in our hospital, we were unable to perform an NK cell activity assay. Therefore, exploration of the etiology of sHLH relies mainly on comprehensive evaluation of clinical manifestations, laboratory findings, bone marrow examination, tissue biopsy, imaging and PET/CT. None of the patients were using or had stopped immunosuppressive therapy at the time of initial HLH diagnosis. 66.7% of the patients had been previously treated with immunosuppressive therapy (corticosteroids) before the initial diagnosis of HLH.

### Follow-up and endpoints

Patient survival was confirmed by telephone interviews and outpatient or inpatient record review. Overall survival (OS) time was defined as the time interval (in days) from the date of diagnosis to the date of death or follow-up cutoff date. The cutoff for follow-up in this study was 31 December 2024, or the date of patient death or loss to follow-up. Three patients were lost to follow-up, and the survival status of the remaining patients was clearly documented.

### Blood sample collection

Two milliliters of fasting venous blood was collected from the study subjects early in the morning in EDTA anticoagulation tubes. The serum was centrifuged at 1800 × g for 25 min within 30 min after collection, separated and immediately frozen at −80°C. The samples were thawed and mixed well before testing.

### Biochemical testing

Serum S100A8/9 levels were measured with an enzyme-linked immunosorbent assay (ELISA) kit (S100A8/9; Proteintech, Wuhan Sanying, China). The assay is a quantitative sandwich enzyme immunoassay: the sample is pipetted into an enzyme-labeled strip precoated with a monoclonal antibody specific for the human S100A8/9 heterodimer and incubated at 37°C for 2 h. Subsequently, an enzyme-linked monoclonal antibody against the human S100A8/9 heterodimer was added, and the mixture was incubated for 1 h. After that, HRP-labeled streptavidin was added and incubated for 40 min. Finally, color development solution and termination solution were added sequentially. All operations were performed in strict accordance with the kit instructions. The optical density (OD) of each well was measured at 450 nm, with 630 nm used as the calibration wavelength. The concentration of S100A8/9 in the samples was calculated from the standard curve. Three replicate wells were set up for each sample, and the test was repeated twice to ensure the accuracy of the results.

### Statistical analysis

All the statistical analyses and graphs were performed via SPSS 28.0, R 4.2.2 and GraphPad Prism 9.0. Normally distributed continuous variables are expressed as the mean ± standard deviation (mean ± SD), and nonnormally distributed continuous variables are expressed as the median (M) and interquartile range (IQR); categorical variables are expressed as the number of cases (n) and percentage (%). Comparisons between groups were made using the independent samples t-test (for normally distributed data) or the Mann‒Whitney U test (for nonnormally distributed data). Categorical variables were tested through chi-square tests.

Spearman’s correlation analysis was used to assess the correlation between the variables; a subject operating characteristic (ROC) curve was constructed, and the area under the curve (AUC), sensitivity and specificity were calculated to evaluate the diagnostic value of serum S100A8/9 levels. Restricted cubic spline (RCS) curves were used to determine the optimal categorization thresholds for S100A8/9 levels. Survival was estimated by Kaplan‒Meier curves, and log-rank tests were used for between-group comparisons. Variables with *P < 0.05* in univariate analyses were included in a multivariate Cox proportional risk regression model to assess the independent predictive value of S100A8/9 levels for the prognosis of patients with sHLHa. All the statistical tests were two-sided, and *P < 0.05* was considered a statistically significant difference.

## Results

### Analysis and comparison of baseline characteristics

Forty-eight patients with sHLHa were ultimately included in this study, all of whom met the diagnostic criteria of HLH-2004 and HScore>169 (Table 1). Thirty-two patients had lymphoma-associated sHLH (LHLH, 66.7%), specifically 10 with NK/T-cell lymphomas, 9 with T-cell lymphomas/leukocytes, 9 with B-cell lymphomas, 1 with peripheral T-cell lymphoma, 2 with diffuse large B-cell lymphomas, and 1 with chronic lymphocytic leukemia. The remaining 16 patients had nonlymphoma-associated sHLH (non-LHLH, 33.3%), of which 10 had infection-associated sHLH (IHLH) and 6 had rheumatologic immune-associated sHLH (AHLH).

**TABLE 1:** Baseline characteristics of 48 patients with sHLH.

Characteristics	AHLH (N = 6)	IHLH (N = 10)	LHLH (N = 32)
Sex (M/F)	3/3	7/3	21/11
Age (years)	66.5 ± 12.05	45.80 ± 16.15	54.53 ± 16.25
Fever^a^, n (%)	6 (100)	10 (100)	28 (88)
Splenomegaly, n (%)	4 (67)	9 (90)	27 (85)
Leucocyte (x10^9^/L)	3.24 (2.05, 4.79)	2.78 (1.02, 7.97)	0.75 (0.51, 3.91)
Hemoglobin (g/L)	84 ± 9.14	102.10 ± 20.79	90.03 ± 24.80
Platelet (x10^9^/L)	59 (41,71)	49 (18, 139)	35 (11, 63)
Fibrinogen (g/L)	2.76 (1.71, 7.66)	2.86 (2.41, 3.46)	1.31 (1.17, 1.79)
Triglyceride (mmol/L)	1.51 (0.91, 2.67)	1.86 (1.33, 2.02)	2.01 (1.70, 3.07)
Ferritin (U/L)	2,242.65 (535.90, 11,244.65)	4,711.3 (1,350.3, 20,000)	4,760.1 (1,151.7, 20,000)
sCD25 (ng/L)	28,825 (12,174.25, 45,276.25)	12,884 (3,749, 17,809)	26,194 (7,317, 32,261)
Hemophogcytic^b^, n (%)	4 (67)	7 (70)	28 (88)
S100A8/9 (µg/mL)	0.50 (0.04, 1.13)	1.49 (0.59, 2.11)	7.58 (1.32, 10.41)

Note: The error in the parameters within the table represents the standard deviation of the mean. The percentages in the table represent the proportion of the total number of people and represent the corresponding numerical ranges.

Abbreviations: sHLH, adult secondary hemophagocytic lymphohistiocytosis; AHLH, immune-related hemophagocytic lymphohistiocytosis; IHLH, infection-associated hemophagocytic lymphohistiocytosis; LHLH, lymphoma-associated hemophagocytic lymphohistiocytosis; sCD25, soluble interleukin–2, receptor.

^a^ Fever means body temperature >38.5°C for more than 7 days.

^b^ Hemophogcytic means Hemophagocytic cells found in bone marrow.

**FIGURE 1:**
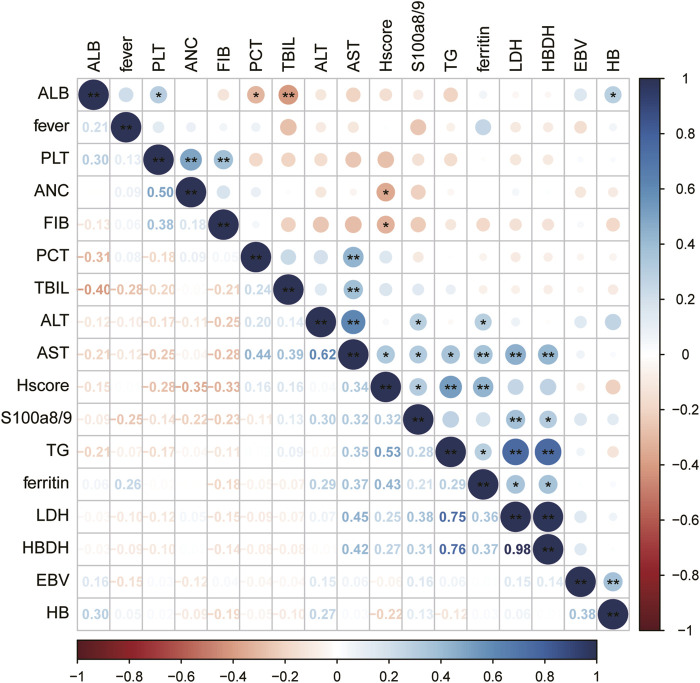
Correlation between variables: Significance of correlation between variables is indicated by “*”for *P < 0.05* and “**” for *P < 0.01*, the darker the color, the stronger the correlation.

Further analysis revealed that the serum S100A8/9 levels in patients with sHLHa were significantly correlated with several laboratory markers (Figure 1). S100A8/9 levels were significantly positively correlated (*P < 0.05*) with alanine aminotransferase (ALT, r = 0.296), aspartate aminotransferase (AST, r = 0.324), lactate dehydrogenase (LDH, r = 0.379), alpha hydroxybutyric acid dehydrogenase (α-HBDB, r = 0.317), adenosine deaminase (ADA, r = 0.445), and HScore (r = 0.318), but had no significant correlations (*P > 0.05*) with other laboratory indices (e.g., fibrinogen, triglycerides, and serum ferritin) (Figure 1).

### Comparison of serum S100A8/9 levels between different groups of patients with sHLHa

#### Comparison of sHLHa patients and healthy controls

**FIGURE 2:**
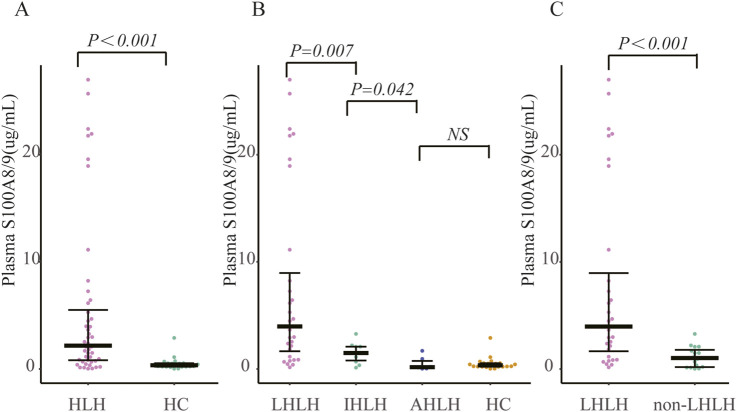
Comparison of S100A8/9 levels in patients with secondary hemophagocytic lymphohistiocytosis (sHLH) vs. healthy controls and lymphoma patients: **(A)** Comparison of S100A8/9 levels in 48 patients with sHLH vs. 21 healthy controls; **(B)** Grouping of patients with sHLH according to different aetiologies and comparison of S100A8/9 levels between the groups; **(C)** Comparison of S100A8/9 levels between 32 patients with LHLH vs. S100A8/9 levels in 16 patients with non-LHLH. Note: AHLH is rheumatology-associated HLH, IHLH is infection-associated HLH, LHLH is lymphoma-associated HLH, and non-LHLH is non-lymphoma-associated HLH.

Serum S100A8/9 levels were significantly higher in sHLHa patients (n = 48) than in healthy controls (n = 21) [0.77 (0.15–2.18) vs. 0.35 (0.21–0.54) µg/mL, *P < 0.001*] (Figure 2A).

#### Comparisons among different etiologic groups

Patients in the LHLH group (n = 32) had significantly higher S100A8/9 levels than did those in the IHLH group (n = 10) [7.58 (1.32–10.41) vs. 1.49 (0.59–2.11) µg/mL, *P < 0.01*]; serum S100A8/9 levels were significantly higher in the IHLH group than in the AHLH group (n = 6) [1.49 (0.59–2.11) vs. 0.50 (0.04–1.13) µg/mL, *P < 0.05*]. However, the difference between the AHLH group and the healthy control group was not statistically significant (*P > 0.05*) (Figure 2B).

#### Comparison of LHLH and non-LHLH

The S100A8/9 levels in the LHLH group (n = 32) were significantly greater than those in the non-LHLH group (n = 16) [7.58 (1.32–10.41) vs. 1.12 (0.60–1.64) µg/mL, *P < 0.05*] (Figure 2C).

### Predictive value of serum S100A8/9 levels for the diagnosis of sHLHa

#### Diagnostic value of the sHLHa score

**FIGURE 3:**
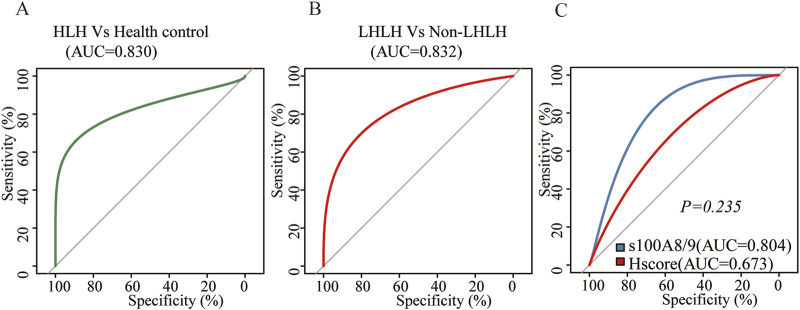
**(A)** ROC curve of S100A8/9 for the comparison between sHLH patients (n = 48) vs. normal people (n = 21); **(B)** ROC curve of S100A8/9 for the comparison between LHLH (n = 32) vs. non–LHLH patients (n = 16); **(C)** ROC curve of sHLH patients (n = 48) for the comparison between S100A8/9 levels vs. HScore.

The area under the ROC curve (AUC) between sHLHa patients (n = 48) and healthy controls (n = 21) was 0.830 (*P < 0.001*), and the optimal threshold value of 0.730 μg/mL resulted in a diagnostic sensitivity of 77.08% and a specificity of 90.48% (Figure 3A).

#### Diagnostic value of LHLH and non-LHLH

ROC curve analysis of the LHLH group (n = 32) and the non-LHLH group (n = 16) revealed an AUC of 0.832 (*P < 0.001*), with a diagnostic sensitivity of 68.75% and specificity of 93.75% at an optimal threshold of 2.2 μg/mL (Figure 3B).

#### Comparison of S100A8/9 with other diagnostic indicators

Further comparison of the diagnostic value of S100A8/9 and HScore for sHLHa showed that both had similar efficacy in diagnosing sHLHa (AUC = 0.804 vs. AUC = 0.673, *P > 0.05*) (Figure 3C).

### Predictive value of the serum S100A8/9 level for the survival and prognosis of patients with sHLH

#### Survival analysis

**FIGURE 4:**
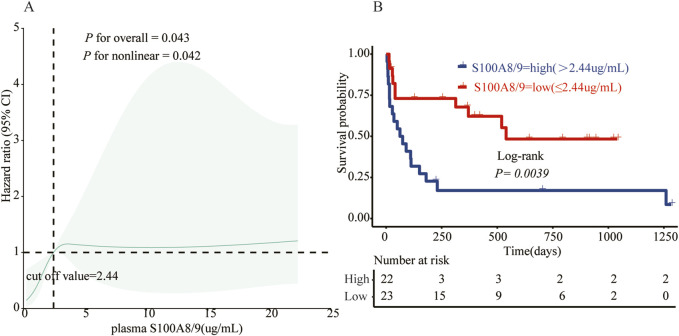
**(A)** The relationship between S100A8/9 and status in 45 sHLH patients was analyzed using a restricted cubic spline regression model. The data were fitted using a restricted cubic spline Cox proportional risk regression model adjusted for sex, age, and ferritin according to the S100A8/9 adjusted state risk ratio of 0.809, with the model 4–noded at the 5th, 35th, 65th, and 95th percentiles for S100A8/9 0.809 (reference value of 2.44). Solid lines indicate HRs and shaded shapes indicate 95% CIs. HR, hazard ratio; CI, confidence interval; **(B)** Survival analysis of the high S100A8/9 expression group (>2.44 μg/mL, n = 22) vs. the low S100A8/9 expression group (≤2.44 μg/m, n = 23) in patients with sHLH.

As of 31 December 2024, of the 48 patients with sHLHa included in this study, three patients with AHLH were lost to follow-up, 29 (64.40%) died, and the median follow-up time for the remaining 16 patients was 790 days (95% confidence interval: 481–1,099 days). The optimal classification threshold for S100A8/9 was determined to be 2.44 μg/mL by RCS curve analysis (Figure 4A). On the basis of this threshold, patients were categorized into the S100A8/9 high-expression group (>2.44 μg/mL, n = 22) and low-expression group (≤2.44 μg/mL, n = 23).

Survival curves were plotted using the Kaplan‒Meier method (Figure 4B), and the results revealed that the risk of death in the S100A8/9 high-expression group was significantly greater than that in the low-expression group. Nineteen patients in the high-expression group (n = 22) died, with a median survival time of 62 days (range: 15–180 days) and a mortality rate of 86.4%. Ten patients in the low-expression group (n = 23) died, with a median survival time of 520 days (range: 301–739 days) and a mortality rate of 43.5%. The difference between the two groups was statistically significant (*p < 0.01*).

**FIGURE 5:**
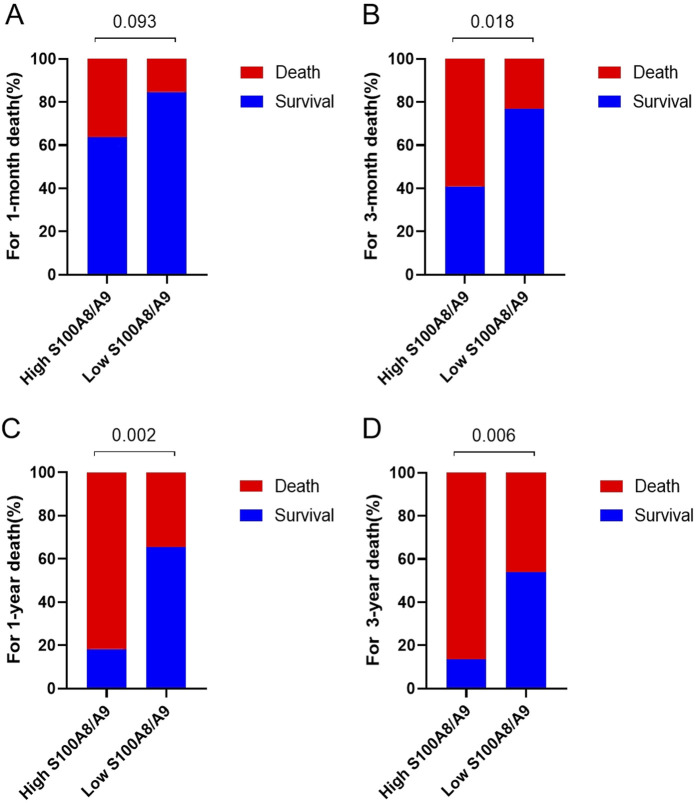
Comparison of mortality rates between high S100A8/9 expression group (>2.44 μg/mL, n = 22) and low S100A8/9 expression group (≤2.44 μg/mL, n = 23). **(A)** Comparison of mortality rates in January, **(B)** comparison of mortality rates in March mortality, **(C)** comparison of mortality rates in 1–year, **(D)** comparison of mortality rates in 3–years mortality.

Further analysis of the survival time of the patients (1 month, 3 months, 1 year and 3 years) revealed that the deaths of the patients in the S100A8/9 high-expression group were mainly concentrated within 3 months (68.4% of the total deaths), whereas the survival rate of the patients decreased significantly after 3 months. In contrast, the deaths of patients in the low-expression group were mainly concentrated within 1 year (70.0% of total deaths), but a certain percentage of patients survived during subsequent follow-up (Figures 5A–D).

#### Risk factor analysis

To explore potential risk factors affecting the prognosis of patients with sHLHa, we performed a univariate analysis of the HLH-2004 diagnostic criteria and other clinical characteristics. The results revealed that a neutrophil count <1x10^9^/L, high S100A8/9 expression, and lymphoma-associated sHLH (LHLH) were risk factors for poor prognosis in patients with sHLHa (*P < 0.05*, Table 2). Variables with *P < 0.05* in the univariate analysis were included in the multivariate Cox proportional risk regression model, which revealed that a neutrophil count < 1x10^9^/L and high S100A8/9 expression were independent risk factors for survival in sHLHa patients (*P < 0.05*, Table 2). Patients with high S100A8/9 expression had a significantly increased risk of death (HR: 3.018, 95% CI: 1.408–6.469).

**TABLE 2:** Univariate and multivariate analysis of overall survival of 45 patients with sHLH.

Variables	Univariate analyses		Multivariate analyses	
	HR (95%CI)	*p*	HR (95%CI)	*p*
Male (female)	1.429 (0.656–3.110)	0.369		
Age (>60 years)	0.706 (0.337–1.480)	0.357		
Fever^a^	0.991 (0.290–3.394)	0.989		
Splenomegaly	1.291 (0.449–3.711)	0.635		
Leucocyte (<1x10^9^/L)	0.342 (0.164–0.712)	0.004	0.366 (0.167–0.800)	0.012
Hemoglobin (<90 g/L)	0.594 (0.291–1.215)	0.154		
Platelet (<100x10^9^/L)	0.185 (0.025–1.359)	0.097		
Fibrinogen (≤1.5 g/L)	0.660 (0.322–1.353)	0.256		
Triglyceride (≥3 mmol/L)	1.439 (0.679–3.051)	0.342		
Ferritin (>500 μg/mL)	0.815 (0.193–3.434)	0.780		
sCD25 (>2400 ng/L)	0.173 (0.019–1.548)	0.117		
Hemophogcytic^b^	1.081 (0.441–2.648)	0.865		
LHLH	0.388 (0.165–0.913)	0.030	0.927 (0.311–2.766)	0.892
S100A8/9 (>2.44 μg/mL)	2.865 (1.362–6.025)	0.006	2.628 (1.056–6.541)	0.038

Note: Use Table 1 to represent the meanings of abbreviations.

Abbreviations: HR, hazard ratio; 95% CI, 95% confidence interval.

^a^ Fever means body temperature >38.5°C for more than 7 days.

^b^ Hemophogcytic means Hemophagocytic cells found in bone marrow.

## Discussion

HLH is a rapidly progressive and highly lethal disease (Nguyen et al., 2024). The diagnostic guidelines for adult sHLHa mostly borrow from those for pediatric pHLH (Griffin et al., 2020). Delayed diagnosis of sHLHa syndrome is significantly associated with increased in-hospital mortality rates (La Rosée et al., 2019). In this study, we evaluated for the first time the diagnostic and prognostic value of serum S100A8/9 in the primary diagnosis of sHLHa. Patients with sHLHa were better differentiated by ROC curves, with a similar diagnostic value to the HScore (*P > 0.05*) and a strong predictive value in identifying different etiologies (*P < 0.01)*. In addition, RCS analysis revealed a nonlinear positive correlation between serum S100A8/9 levels and poor prognosis in adult sHLHa patients, with an inflection point of 2.44 μg/mL, and a significantly shorter survival for patients in the high-level group (median OS: 62 vs. 520 days, P = 0.0039).

In the present study, we found that serum S100A8/9 levels were significantly higher in sHLHa patients than in healthy controls (*p < 0.05*), a result that is consistent with a study by Muhammad S Soyfoo et al. ^17^. S100A8/9, an endogenous alarm protein, plays an important role in a variety of inflammatory diseases and cancers, and its elevated expression level has been widely recognized as a marker of the inflammatory response and disease progression. For example, in systemic lupus erythematosus (SLE), S100A8/9 levels are significantly elevated and correlate with disease activity (Soyfoo et al., 2009). In addition, S100A8/9 has significant proinflammatory effects on a variety of cancers (lung, liver, colorectal, etc.) and inflammatory diseases (sepsis, rheumatoid arthritis, etc.) (Başsorgun et al., 2014; Gebhardt et al., 2015; Hermani et al., 2006; Liu et al., 2022; Petersen et al., 2000; Sy et al., 2005; Zhang et al., 2023; Di Ceglie et al., 2018; Rodriguez-Smith et al., 2021; Wei et al., 2025). Our study found similar efficacy of serum S100A8/9 levels and diagnostic value of Hscore score in terms of diagnosing patients with sHLH (*p > 0.05*). In the present study, S100A8/9 not only effectively distinguished sHLH patients from other healthy individuals but also exhibited differential expression in sHLH patients with different etiologic subtypes (e.g., S100A8/9 levels were higher in LHLH patients than in non-LHLH patients), suggesting that sHLH patients with different etiologies may have different pathophysiologic mechanisms and inflammatory response intensities, which is also consistent with previous studies on the differences in the survival prognoses of sHLH patients with different etiologies (Jongdee et al., 2024).

The results of the present study revealed a significant positive correlation between the S100A8/9 levels of sHLHa patients and their survival (*p < 0.05*) and identified a threshold of 2.44 μg/mL for predicting poor prognosis. Patients had a significantly increased risk of death when their serum S100A8/9 levels exceeded this threshold. This finding is consistent with previous findings (Hou et al., 2023; Jakobsson et al., 2023; Krenkel et al., 2020; R et al., 2021; Su et al., 2022) that elevated S100A8/9 levels are strongly associated with poor prognosis in a variety of inflammatory diseases and malignancies, such as patients with severe COVID-19 and patients with advanced extranodal NK/T-cell lymphoma (ENKL) (Lee et al., 2023; Zhou et al., 2016), where S100A8/9 levels correlate with poor survival. Further multivariate Cox regression analysis confirmed that high S100A8/9 expression was an independent risk factor for poor prognosis in patients with sHLHa syndrome, suggesting its significant predictive value in clinical practice.

We also found that S100A8/9 levels in sHLHa patients were significantly correlated (*P < 0.05*) with liver function indices (ALT, AST, LDH, α-HBDH, ADA). This result is consistent with that of the study by Chang et al. (2024), suggesting that the upregulation of S100A8/9 expression may affect the survival prognosis of patients by exacerbating liver injury and fibrosis. Calcineurin S100A8/9, binds to RAGE, scavenger receptor (CD36), CD33 or Toll-like receptor 4 (TLR4) to control protein phosphorylation, various enzyme activities, Ca2+ homeostasis, NADPH oxidase activation and intermediate filament polymerisation (Möller et al., 2023; Boucher et al., 2024), This association may reflect a pathophysiological mechanism of multiorgan dysfunction in sHLHa patients, especially in the presence of liver damage, which may be further exacerbated by S100A8/9 through the promotion of inflammatory responses and cytokine storms, this may lead to variability in serum S100A8/9 levels in different patients.

Although the exact mechanism of action of S100A8/9 in sHLHa is unclear, we propose a possible hypothesis that lymphocyte expansion and sustained activation of monocyte macrophages may lead to the upregulation of S100A8/9 expression, and this upregulation induces the secretion of proinflammatory cytokines (e.g., TNFα, IL-1β, and IL-6) and chemokines (e.g., MIP-1α and MIP-1α) by monocyte macrophages through the activation of the TLR4/myeloid differentiation Factor 88 (MyD88)/nuclear factor KB (NF-κ B) signaling pathway, which induces monocyte macrophages to secrete proinflammatory cytokines, thereby triggering a cytokine storm leading to multiorgan dysfunction (Yao et al., 2022; Hou et al., 2023; Al-Samkari and Berliner, 2018; Ramos-Casals et al., 2014; Cristaldi et al., 2023; Lu et al., 2024; Xin et al., 2024; Ye et al., 2025; Zheng et al., 2022). In addition, Haijun Wen et al. reported that the V140A mutation in the ECSIT gene activated the NF-κ B pathway by promoting the formation of S100A8/9, which in turn induced macrophage overactivation to secrete cytokines, ultimately leading to phagocytosis syndrome (Wen et al., 2018). These findings further support the critical role of S100A8/9 in the pathogenesis of sHLHa. Recent studies have also shown that the proinflammatory mechanism of S100A8/9 *in vivo* is highly similar to the pathogenesis of HLH characterized by a cytokine storm. For example, Giada Ingoglia et al. reported that macrophage agonistic anti-CD40 antibody treatment induced HLH by establishing a macrophage-centered mouse model (Ingoglia et al., 2020). Fan Su et al. demonstrated that a nanocomplex loaded with the S100A8/9 inhibitor ABR2527 effectively blocked neutrophil-S100A8/9-toll-like receptor (TLRS)-inflammatory vesicle signalling, thereby attenuating sepsis-induced acute lung injury in preclinical mice (Su et al., 2025). Recent BLOOD findings that neutrophil activation triggers elevated associated inflammatory factors in AOSD-MAS patients have elucidated the mechanism by which rucotinib can modulate neutrophil activation through broad inhibition of the JAK-STAT signalling pathway (Ma et al., 2025). Notably, lipid rafts can act as signalling hubs in the vascular system and in the inflammatory response (Warda et al., 2025). Larisa Dubrovsky’s study showed that inhibitors of methyl-β-cyclodextrin, statins, and the lipid raft-associated receptor IGF1R prevented lipopolysaccharide (LPS) from binding to TLR4 to activate NF-κB, which stimulated macrophages to release higher levels of proinflammatory cytokines (induced by extracellular vesicles of HIV- 1 Nef extracellular vesicles induced) (Dubrovsky et al., 2022). Sulfonamides block TLR4-lipid raft co-localisation to inhibit LPS-induced hyperinflammatory responses (Kim et al., 2020). Wang H et al. demonstrated the potential therapeutic value of biocompatible LPS-stimulated macrophage membrane-encapsulated nanoparticles (LMNPs) in the inhibition of HLH (Wang H. et al., 2023). These studies provide a theoretical basis for future interventional therapies targeting S100A8/9 or its downstream pathways.

Although this study provides new insights into the role of S100A8/9 in sHLHa, several limitations remain. First, this study was a single-center, retrospective design with a small sample size, which may have introduced selection bias. Second, the lack of longitudinal data limits the assessment of changes in S100A8/9 dynamics. Finally, the failure to analyze other proinflammatory cytokines or inflammatory markers comprehensively limits the full validation of the S100A8/9 performance profile. Future studies should aim to validate these findings in larger, multicenter prospective cohorts and explore the value of the molecular mechanisms of S100A8/9 in sHLHa and its potential therapeutic targets. In addition, combined analysis with other inflammatory markers or therapeutic targets may further increase the value of S100A8/9 for clinical applications.

## Conclusion

This study demonstrated that S100A8/9 is not only effective in differentiating sHLHa patients from other healthy individuals but also provides important information for etiologic typing and survival prediction. S100A8/9 levels were nonlinearly and positively correlated with poor survival in sHLHa patients, with a significant threshold effect (2.44 μg/mL). Therefore, S100A8/9 is expected to be an important biomarker for the diagnosis, prognostic assessment and personalization of sHLHa.

## Data Availability

The raw data supporting the conclusions of this article will be made available by the authors, without undue reservation.
